# Supplementary material for: Structural basis of tubulin detyrosination by VASH2/SVBP heterodimer
Source: Nat Commun. 2019 Jul 19;10:3212. doi: 10.1038/s41467-019-11277-8 (PMC6642083; doi:10.1038/s41467-019-11277-8)
Supplement: Supplementary file 1 — Supplementary Information [file 41467_2019_11277_MOESM1_ESM.pdf]

Supplementary Information

Structural basis of tubulin detyrosination by VASH2/SVBP heterodimer

C. Zhou, L. Yan *et al*.

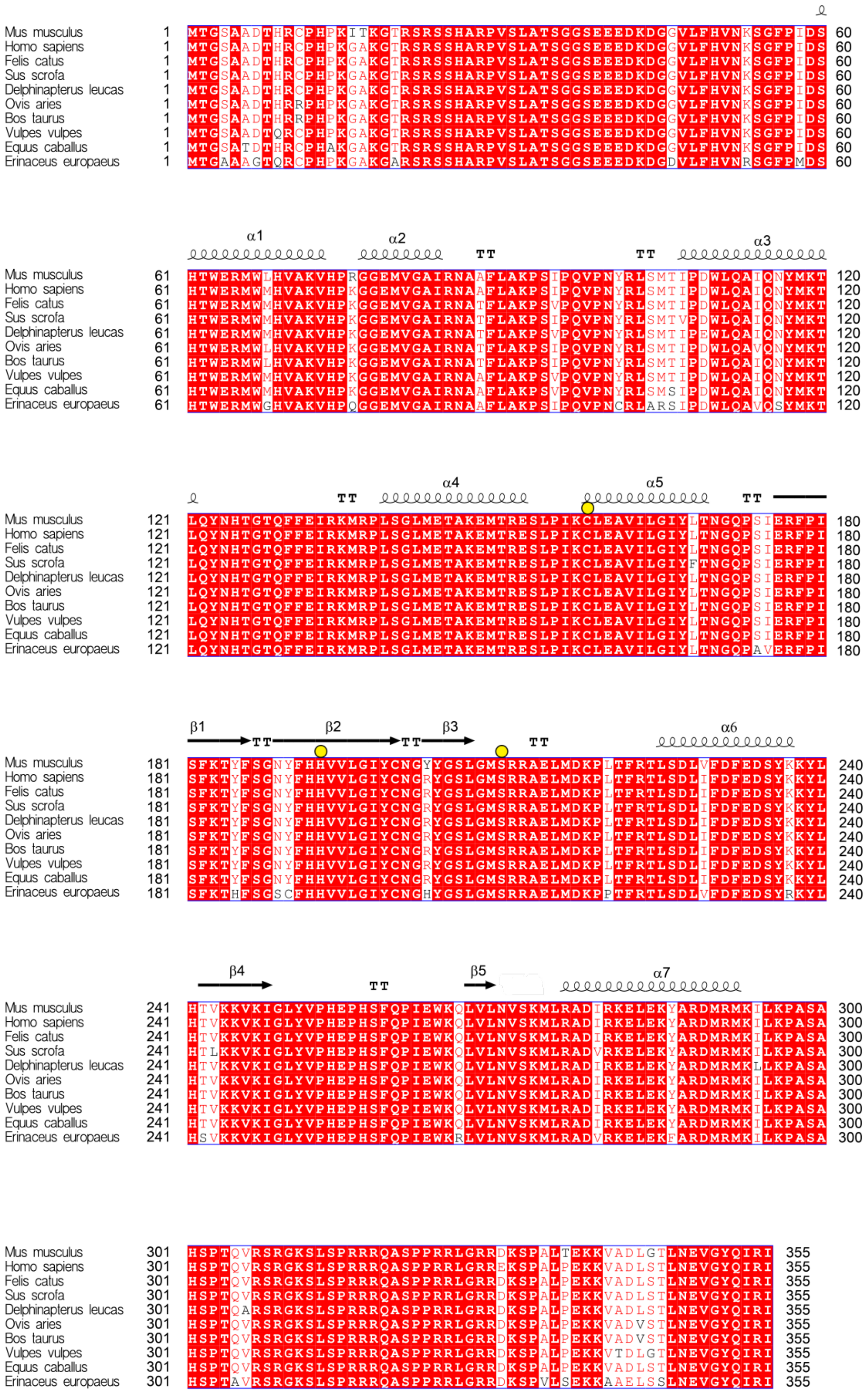

Supplementary Figure 1 | Sequence alignment of VASH2. The sequence of *Mus musculus* VASH2 (UniProt ID: Q8C5G2) is aligned with its homologs from *Homo sapiens* (UniProt ID:

Q86V25), *Felis catus* (UniProt ID: M3X479), Sus scrofa (UniProt ID: F1S2X7), *Delphinapterus leucas* (UniProt ID: A0A2Y9PUA2), *Ovis aries* (UniProt ID: W5PJB2), *Bos Taurus* (UniProt ID: F6PTR8), *Vulpes vulpes* (UniProt ID: A0A3Q7SAI9), *Equus caballus* (UniProt ID: F6VRQ7), and *Erinaceus europaeus* (UniProt ID: A0A1S3AFZ8). No significant similarities are found in microorganisms and plants such as Escherichia coli, Deinococcus radiodurans, *Saccharomyces cerevisiae*, *Oryza sativa Japonica Group* and *Arabidopsis thaliana*. The alignment is generated using the MultAlin and UniProt. Sequence identity is indicated by white letters against a red background, and sequence similarity is indicated by red letters. The secondary elements are labeled at top of the alignment. The Cys-His-Ser catalytic triad is marked with yellow circles at the top.

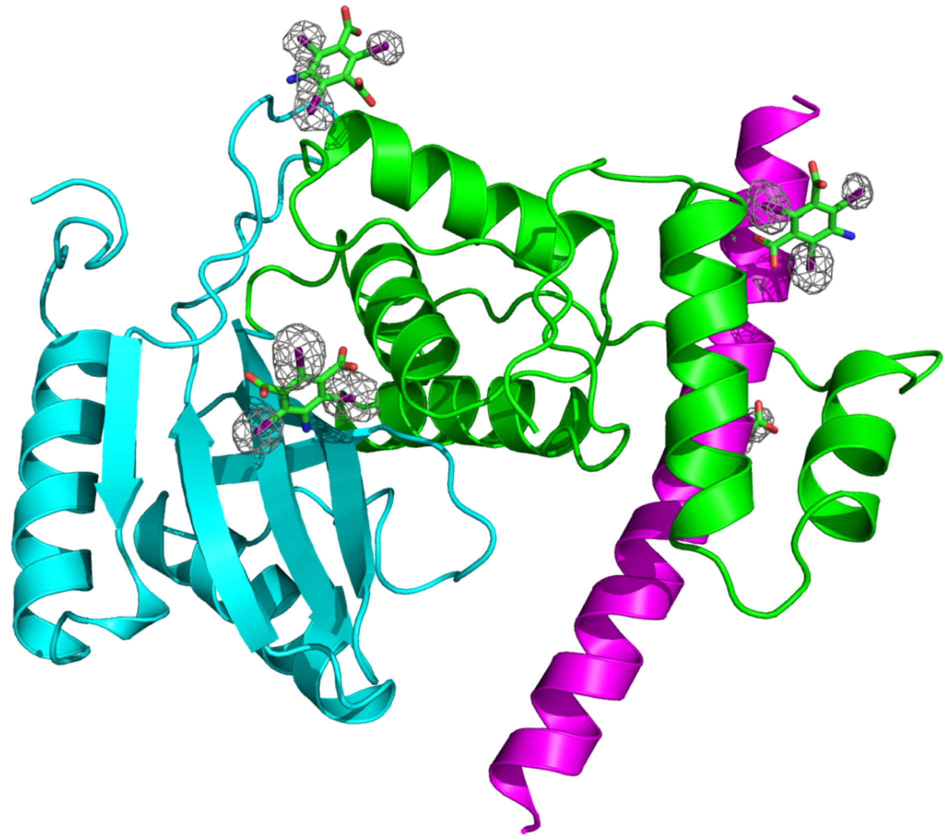

Supplementary Figure 2 | Electron density map of I3C compounds in VASH2/SVBP structure. The map is contoured at 3σ (gray). The equilateral triangle formed by the I atom is clearly visible.

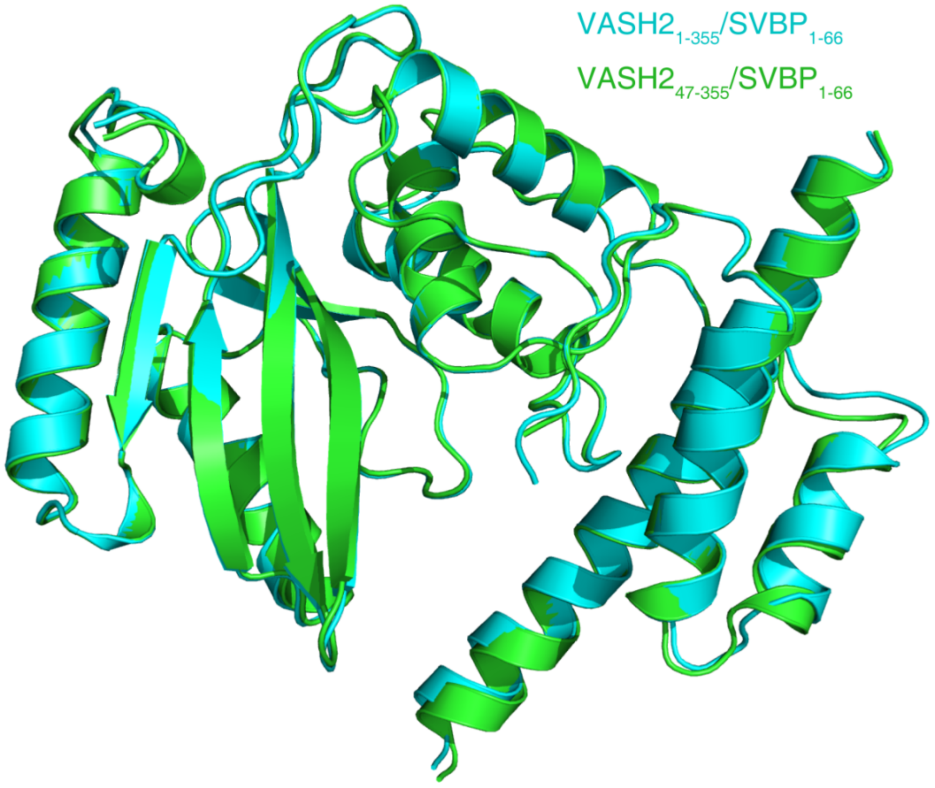

Supplementary Figure 3 | The RMSD between VASH2$_{1\text{-}355}$/SVBP$_{1\text{-}66}$ structure and VASH2$_{47\text{-}355}$/SVBP$_{1\text{-}66}$ structure is 0.28.

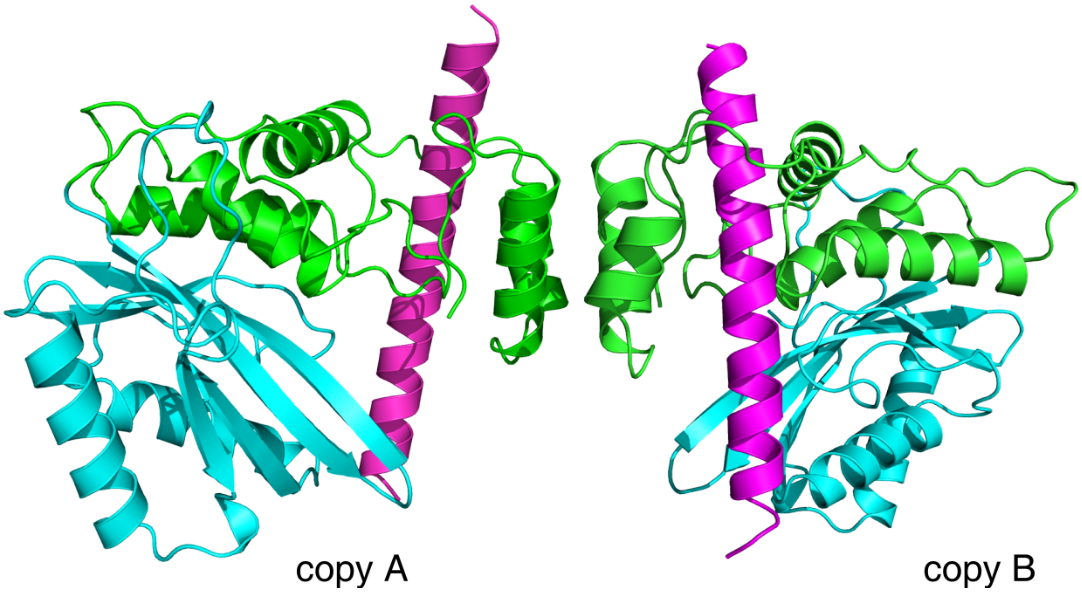

Supplementary Figure 4 | Arrangement of the two copies of VASH2$_{47\text{-}355}$/SVBP$_{1\text{-}66}$ heterodimer in an asymmetric unit.

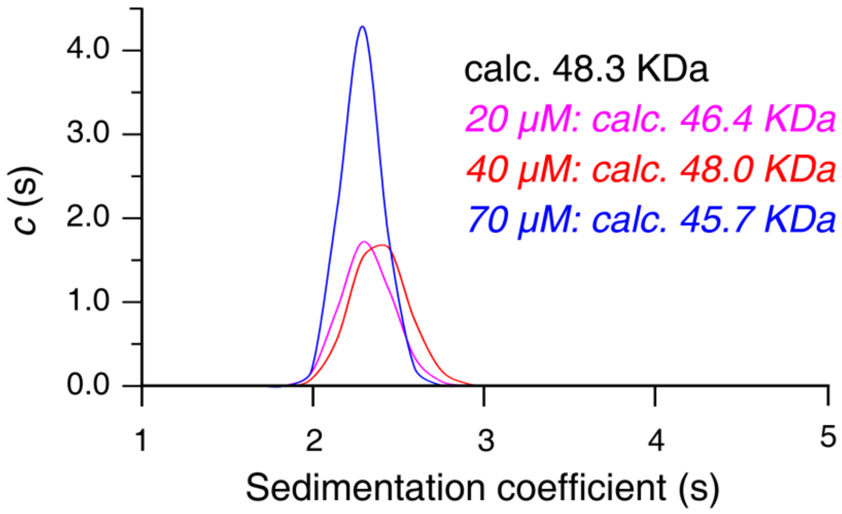

Supplementary Figure 5 | Analytical ultracentrifugation experiments. The experimentally measured molecular weight at different VASH2/SVBP concentration is almost equal to the sum of VASH2 and SVBP (48.3 kDa in total).

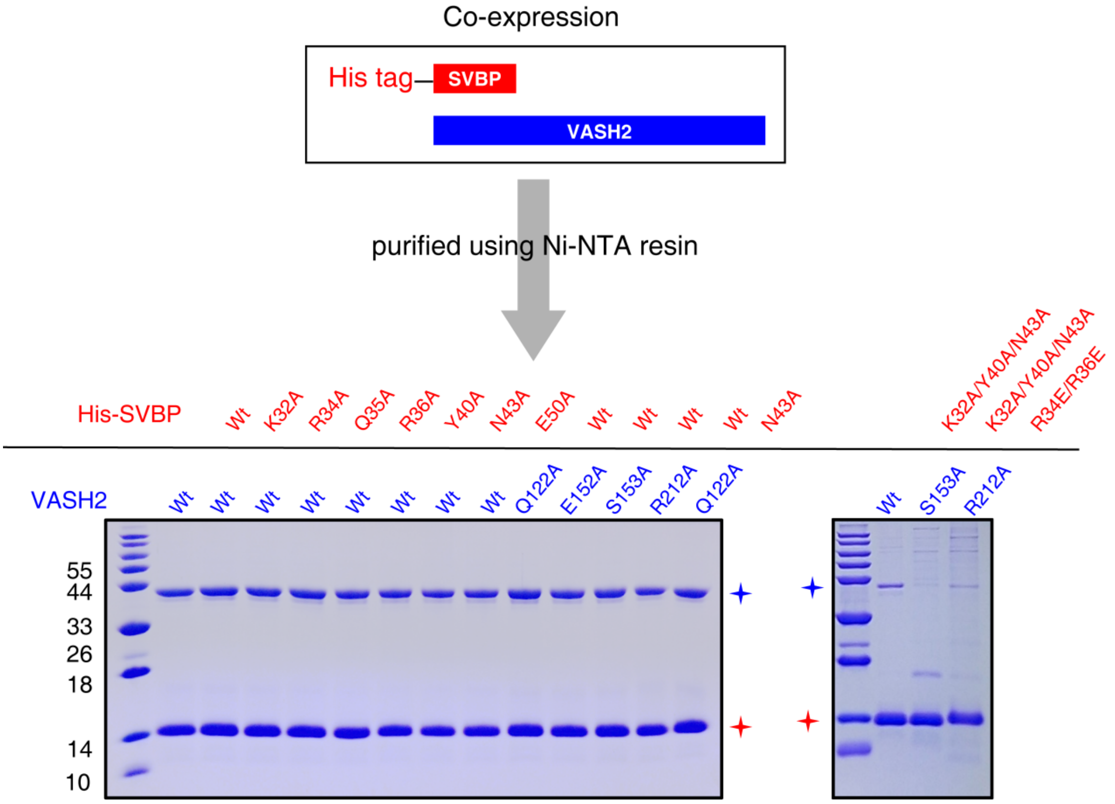

Supplementary Figure 6 | Using a co-expression and purification strategy to characterize the interaction between VASH2 and SVBP. SVBP enhances VASH2 solubility by binding; thus, a weakened interaction would make VASH2 species less soluble during co-expression and purification, as indicated by the attenuated VASH2 bands in Tricine-SDS-PAGE gels. Blue and red start mark the positions of VASH2 and SVBP in the gel, respectively.

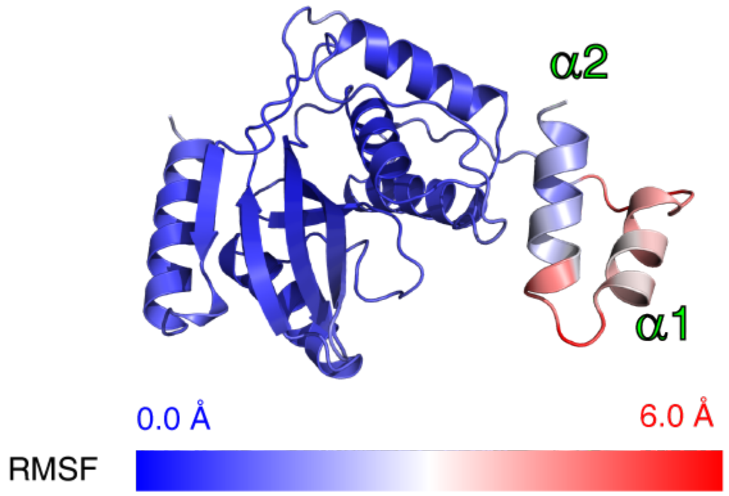

Supplementary Figure 7 | The C_α RMSF of VASH2 in MD simulations without SVBP is displayed in a scale from blue (0.0 Å) to red (6.0 Å). Flexible N- and C-terminal loops are omitted for better visualization. The induced flexible regions upon SVBP detachment are mainly located in the α1- and α2- helices.

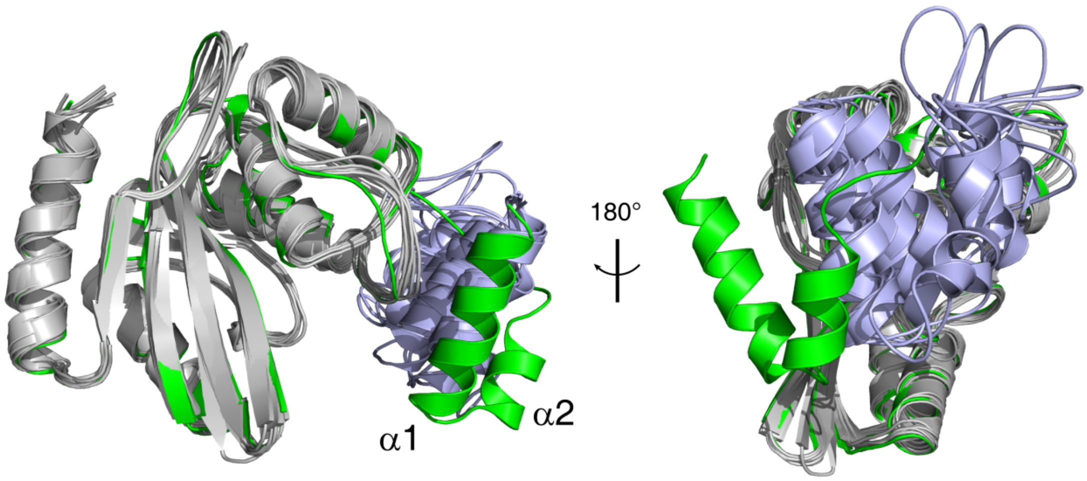

Supplementary Figure 8 | The α1- and α2- helices of VASH2 fluctuate integrally and maintain their helical fold during MD simulations. Structures at 1 ns, 50 ns, 100 ns, 150 ns, 200 ns, 250 ns, 300 ns, 350 ns and 400 ns during MD simulations are selected to represent protein dynamics. The VASH2 initial structure for the simulations (extracted from VASH2/SVBP crystal structure) is colored green, and the α1- and α2- helices of the simulated structures are colored light blue with other parts in gray.

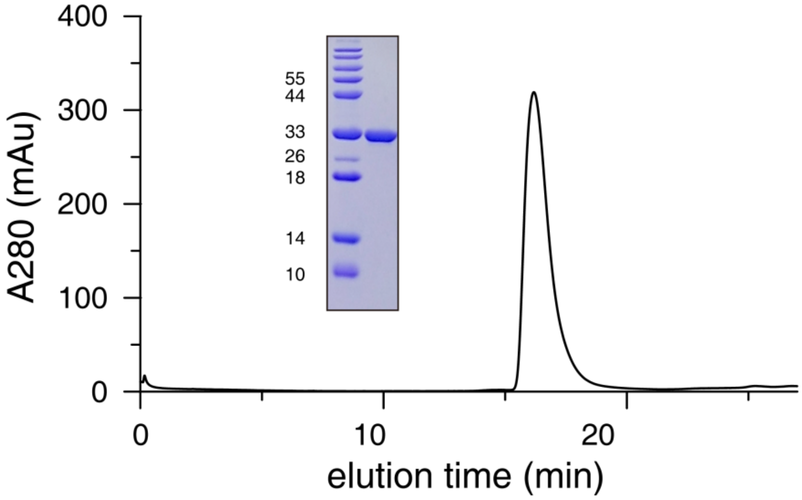

Supplementary Figure 9 | VASH2_Δα1/α2 is soluble and is well behaved. The C-terminal Flag tagged VASH2_Δα1/α was expressed in *sf9* cells and was purified using Flag-affinity column, heparin sepharose column in tandem. The behavior was analyzed using SEC 650 column.

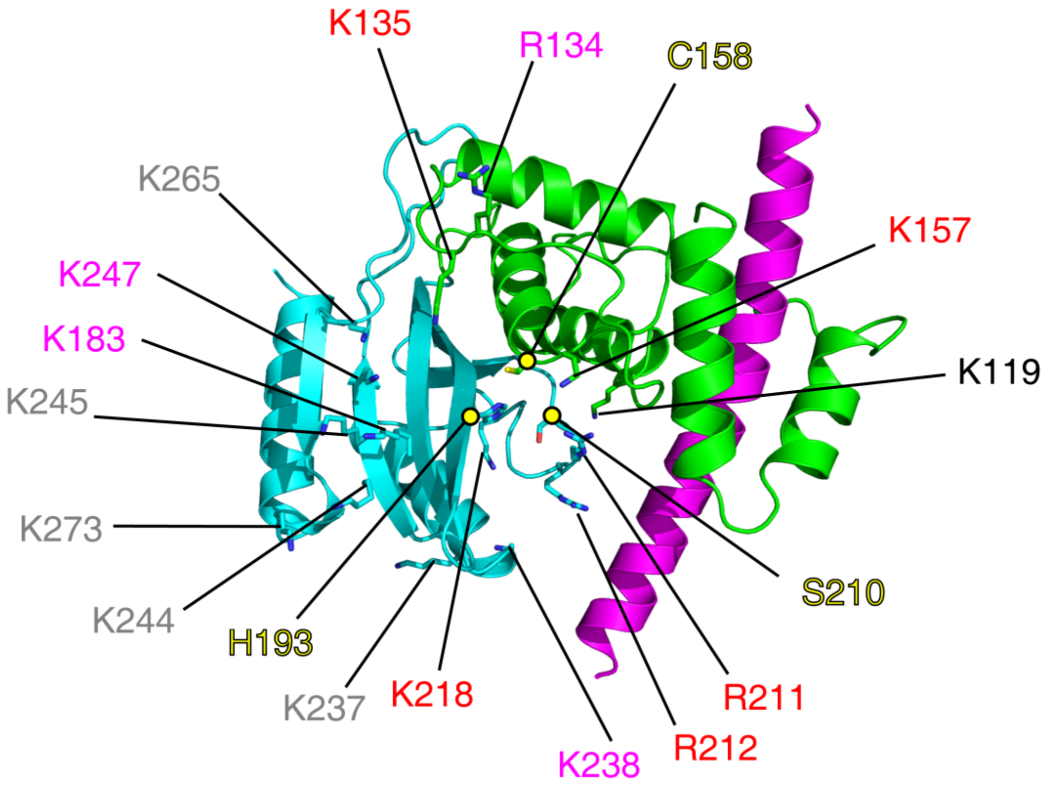

Supplementary Figure 10 | Cartoon representation of Fig. 4a in main text. The NTD and CTD of VASH2 are colored green and cyan, respectively. SVBP is colored magenta.

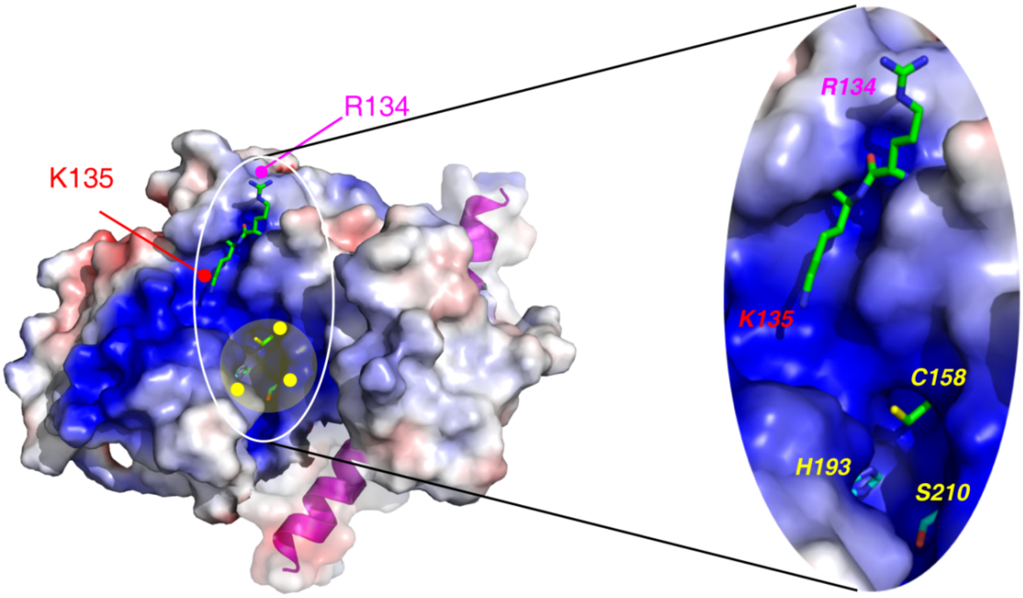

Supplementary Figure 11 | Side chain orientations of R134 and K135 in the positively charged groove on VASH2/SVBP. R134, K135 and residues in the catalytic triad are shown in stick representation and highlighted.

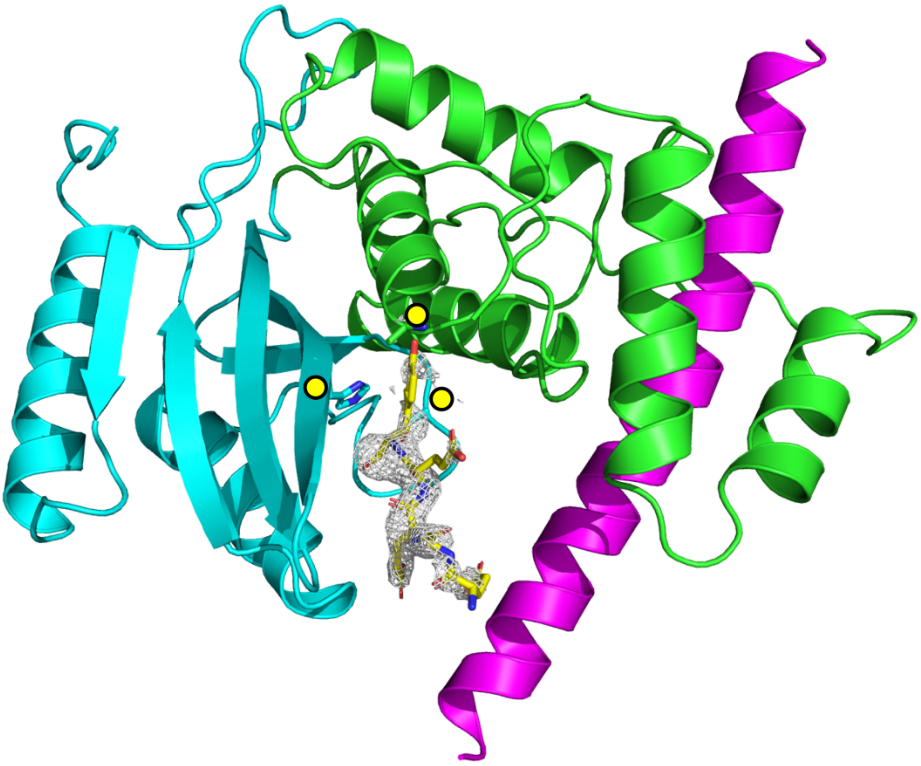

Supplementary Figure 12 | Ternary structure of tubulin tail/VASH2/SVBP. The 2Fo-Fc electron density of EGEEY peptide is contoured at 1σ in gray. The peptide is shown in stick representation in yellow. The Cys-His-Ser catalytic triad is shown in stick representation and highlighted.

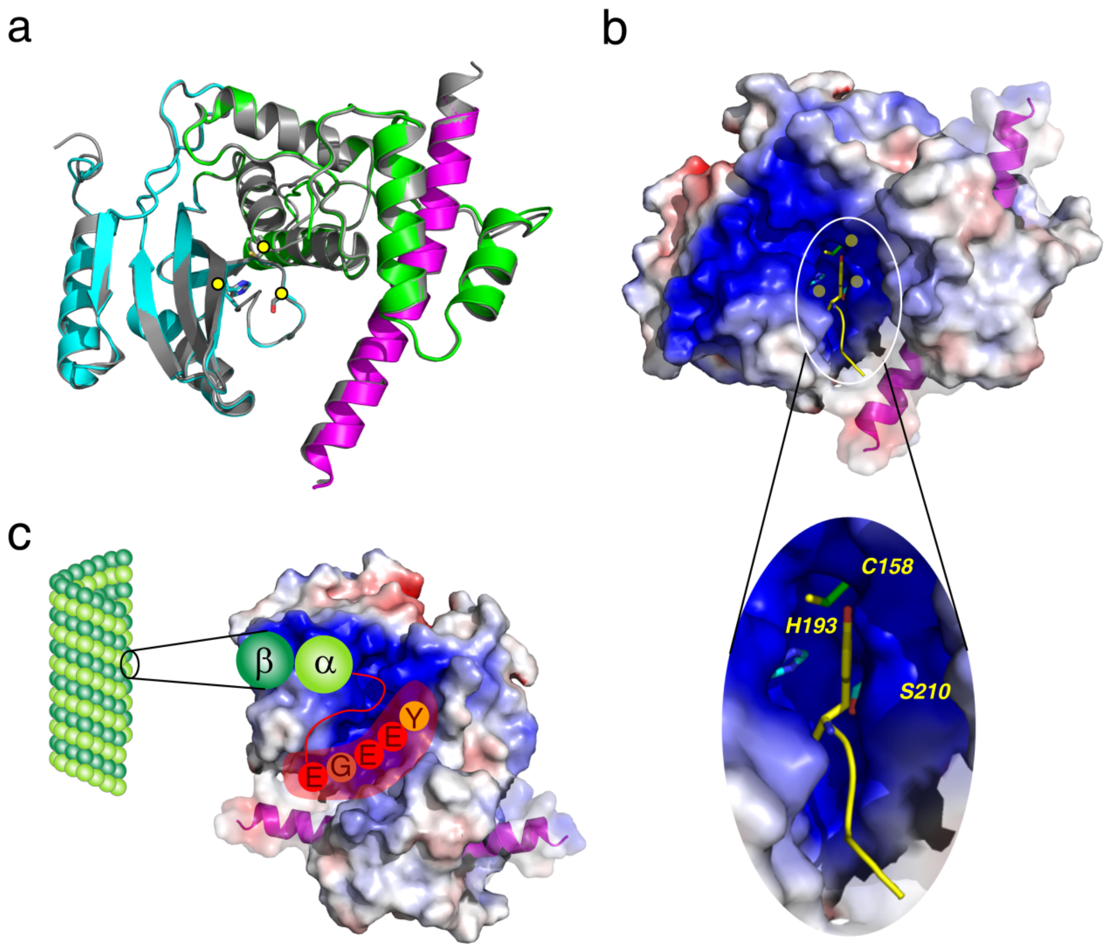

Supplementary Figure 13 | Mechanism of tubulin tail recognition by VASH2/SVBP heterodimer. (a) Structural alignment of the VASH2/SVBP heterodimer (gray cartoon) and VASH/SVBP/tail complex (green NTD, cyan CTD, and magenta SVBP). The Catalytic residues are shown in stick representation, and the tail is omitted for superposition. **(b)** Surface visualization shows that the tail is pre-arranged in the positive groove for tyrosine cleavage at the catalytic pocket. The C-terminal tyrosine is shown in stick representation in yellow, and other parts are shown in cartoon representation. **(c)** A model of VASH2/SVBP targeting α-tubulin for detyrosination. The glutamate-rich tail inserts into the positive groove near the catalytic pocket, and other parts of the positive groove may be responsible for the interactions with tubulin folded domains and microtubule lattice.

Supplementary Table 1. Statistics of data collection and refinement.

structure	$VASH2_{47\text{-}355}/SVBP_{1\text{-}66}$ (*apo*)	$VASH2_{1\text{-}355}(C158A)/SVBP_{1\text{-}66}$ /peptide (EGEEY-boud)	$VASH2_{1\text{-}355}/SVBP_{1\text{-}66}$ /I3C (I3C-bound)
PDB identifier	6JZC	6JZD	6JZE
**Data collection**			
Space group	$C222_{1\text{1.}}$	$C222_1$	$C222_1$
Wavelength	0.9789	0.9791	1.5418
Cell dimensions			
a, b, c (Å)	81.80, 120.93, 193.19	74.00, 111.34, 126.32	72.69, 111.11, 126.45
α, β, γ (°)	90.0, 90.0, 90.0	90.0, 90.0, 90.0	90.0, 90.0, 90.0
Resolution (Å)	45-2.20 (2.27-2.20)	45-2.48 (2.58-2.48)	45-2.51 (2.60-2.51)
R_{merge} (%)	9.8(82.1)	5.9(75.9)	9.3(42.1)
R_{pim} (%)	4.1(34.6)	2.5(33.2)	1.8(9.6)
$CC_{1/2}$ (%)	99.8(89.5)	99.9(76.4)	99.5(97.6)
I / σI	12.9(2.6)	17.1(2.4)	53.8(9.3)
Completeness (%)	99.7(98.8)	99.6(98.5)	100.0(100.0)
No. measured reflections	323,326	119,799	457,680
No. unique reflections	48,774	18,801	17,849
Redundancy	6.6(6.5)	6.4(6.1)	25.6(18.7)
Wilson B factor ($Å^2$)	33.35	60.7	38.73
Anomalous correlation			0.394
Anomalous redundancy			9.7
Number of sites			12
**Refinement**			
R_{work} /R_{free} (%)	18.09/21.48	17.93/22.96	22.30/26.77
No. atoms			
Protein main chain	2,288	1,144	1,156
Protein side chain	2,424	1,215	1,223
Protein all atoms	4,712	2,359	2,379
Water molecules	260	16	73
Other entities	48	43	64
All atoms	5,020	2,418	2,516
B-factors ($Å^2$)			
Protein main chain	43.3	69.7	46.9
Protein side chain	48.9	75.6	51.7

Protein all atoms	46.2	72.6	49.3
Water molecules	49.0	61.0	48.9
Other entities	58.7	125.8	157.0
All atoms	46.5	73.6	52.1
R.m.s. deviations			
Bonds (Å)	0.007	0.008	0.011
Angle (°)	0.851	0.943	1.321
Ramachandran plot statistics (%)			
Most favorable	97.0	97.9	96.8
Additionally allowed	3.0	2.1	3.2
Disallowed	0	0	0

Values in parentheses are for the highest resolution shell. $R_{merge}=\Sigma_h\Sigma_i|I_{h,i}-I_h|/\Sigma_h\Sigma_i I_{h,i}$, where I_h is the mean intensity of the *i* observations of symmetry related reflections of *h*. $R=\Sigma|F_{obs}-F_{calc}|/\Sigma F_{obs}$, where F_{calc} is the calculated protein structure factor from the atomic model (R_{free} was calculated with 5% of the reflections selected).

Supplementary Table 2. Particular residues in polar interaction between VASH2 and SVBP

VASH2 (atom)	SVBP (atom)	Distance (Å)	Polar interaction
Ile 58 (O)	Gln 35(NE2)	2.97	hydrogen bound
Ile 58 (N)	Gln 35(OE1)	2.96	hydrogen bound
Ile 84(O)	Arg 34(NH2)	3.38	hydrogen bound
Arg 85(O)	Arg 34(NH2)	3.09	hydrogen bound
Ala 87(O)	Arg 34(NH1)	2.84	hydrogen bound
Ala 87(O)	Arg 34(NH2)	2.75	hydrogen bound
Pro 93(O)	Arg 36(NH2)	2.72	hydrogen bound
Ser 94(O)	Arg 36(NH1)	2.94	hydrogen bound
Leu 121(O)	Tyr 40(OH)	2.65	hydrogen bound
Gln 122(OE1)	Asn 43(ND2)	2.89	hydrogen bound
Try 123(O)	Asn 43(ND2)	2.88	hydrogen bound
His 125(N)	Asn 43(OD1)	2.69	hydrogen bound
Thr 126(N)	Asn 43(OD1)	3.70	hydrogen bound
Ser 153(O)	Arg 36(NE)	2.79	hydrogen bound
Ser 153(OG)	Arg 36(NH1)	3.79	hydrogen bound
Ser 153(O)	Tyr 40(OH)	3.37	hydrogen bound
Arg 212(NH1)	Glu 50(OE1)	3.12	hydrogen bound
Arg 212(NH2)	Glu 50(OE2)	2.89	hydrogen bound
Glu 152(OE1)	Lys 32(NZ)	3.81	Salt bridges
Glu 152(OE2)	Lys 32(NZ)	3.10	Salt bridges
Arg 212(NH1)	Glu 50(OE1)	3.12	Salt bridges
Arg 212(NH2)	Glu 50(OE1)	3.73	Salt bridges
Arg 212(NH1)	Glu 50(OE2)	3.75	Salt bridges
Arg 212(NH2)	Glu 50(OE2)	2.89	Salt bridges

The residue interactions between VASH2 and SVBP were analyzed using the PDBe PISA web service.

Supplementary Table 3. Primers used in this study

Primers	Primer sequences (5′–3′)
**PCR products were cloned into pFastBac1 vector**	
VASH2$_{1\text{-}355}$-Fw	GAATTCCATATGACCGGCTCCGCCGCTGATACC
VASH2$_{1\text{-}355}$-Rv	GCCCTCGAGTTAGATCCTGATCTGGTAGCCCACCT
VASH2$_{47\text{-}355}$-Fw	GAATTCCATATGGTGCTGTTCCACGTGAACAAGTCCG
VASH2$_{47\text{-}355}$-Rv	GAATTCCATATGTCCATCCCCCAGGTGCCCAAC
SVBP$_{1\text{-}66}$-Fw	GAATTCCATATGGACCCTCCTGCCAGAAAGGAGAAGAG
SVBP$_{1\text{-}66}$-Rv	GCCCTCGAGTTACTCGCCGGGGGGCTGCATCTGCTTACAAAACT
VASH2$_{1\text{-}355}$_C158A-Fw	CTGCCTATCAAGGCTCTGGAGGCCGTG
VASH2$_{1\text{-}355}$_C158A-Rv	CACGGCCTCCAGAGCCTTGATAGGCAG
VASH2$_{1\text{-}355}$_H193A-Fw	CAATTACTTTCACGCCGTGGTGCTGG
VASH2$_{1\text{-}355}$_H193A-Rv	CCAGCACCACGGCGTGAAAGTAATTG
VASH2$_{1\text{-}355}$_S210A-Fw	TGGGCATGGCCAGGAGGGCCGAGCTGA
VASH2$_{1\text{-}355}$_S210A-Rv	TCAGCTCGGCCCTCCTGGCCATGCCCA
VASH2$_{1\text{-}355}$_K119E-Fw	AATTACATGGAGACCCTGCAG
VASH2$_{1\text{-}355}$_K119E-Rv	CTGCAGGGTCTCCATGTAATT
VASH2$_{1\text{-}355}$_R134E-Fw	TTCGAGATCGAGAAGATGAGG
VASH2$_{1\text{-}355}$_R134E-Rv	CCTCATCTTCTCGATCTCGAA
VASH2$_{1\text{-}355}$_ K135E-Fw	GAGATCAGGGAGATGAGGCCT
VASH2$_{1\text{-}355}$_ K135E-Rv	AGGCCTCATCTCCCTGATCTC
VASH2$_{1\text{-}355}$_ K157E-Fw	CTGCCTATCGAGTGTCTGGAG
VASH2$_{1\text{-}355}$_ K157E-Rv	CTCCAGACACTCGATAGGCAG
VASH2$_{1\text{-}355}$_ K183E-Fw	ATCAGCTTCGAGACATACTTC
VASH2$_{1\text{-}355}$_ K183E-Rv	GAAGTATGTCTCGAAGCTGAT
VASH2$_{1\text{-}355}$_ R211E-Fw	GGCATGTCCGAGAGGGCCGAG
VASH2$_{1\text{-}355}$_ R211E-Rv	CTCGGCCCTCTCGGACATGCC
VASH2$_{1\text{-}355}$_ R212E-Fw	ATGTCCAGGGAGGCCGAGCTG
VASH2$_{1\text{-}355}$_ R212E Rv	CAGCTCGGCCTCCCTGGACAT
VASH2$_{1\text{-}355}$_ K218E-Fw	CTGATGGATGAGCCTCTGACC
VASH2$_{1\text{-}355}$_ K218E-Rv	GGTCAGAGGCTCATCCATCAG
VASH2$_{1\text{-}355}$_ K237E-Fw	GATTCCTACGAGAAGTACCTG
VASH2$_{1\text{-}355}$_ K237E-Rv	CAGGTACTTCTCGTAGGAATC
VASH2$_{1\text{-}355}$_ K238E-Fw	TCCTACAAGGAGTACCTGCAC
VASH2$_{1\text{-}355}$_ K238E-Rv	GTGCAGGTACTCCTTGTAGGA
VASH2$_{1\text{-}355}$_ K244E-Fw	CACACCGTGGAGAAGGTGAAG
VASH2$_{1\text{-}355}$_ K244E-Rv	CTTCACCTTCTCCACGGTGTG
VASH2$_{1\text{-}355}$_ K245E-Fw	ACCGTGAAGGAGGTGAAGATC
VASH2$_{1\text{-}355}$_ K245E-Rv	GATCTTCACCTCCTTCACGGT
VASH2$_{1\text{-}355}$_ K247E-Fw	AAGAAGGTGGAGATCGGCCTG
VASH2$_{1\text{-}355}$_ K247E-Rv	CAGGCCGATCTCCACCTTCTT

VASH2$_{1-355}$_ K265E-Fw	ATCGAGTGGGAGCAGCTGGTG
VASH2$_{1-355}$_ K265E-Rv	CACCAGCTGCTCCCACTCGAT
VASH2$_{1-355}$_ K273E-Fw	AATGTGTCCGAGATGCTGAGG
VASH2$_{1-355}$_ K273E-Rv	CCTCAGCATCTCGGACACATT
**PCR products were cloned into pFastBacDual vector**	
VASH2$_{1-355}$-Fw	GCCCTCGAGATGACCGGCTCCGCCGCTGATACCCACA
VASH2$_{1-355}$-Rv	CGGGGTACCTTAGATCCTGATCTGGTA
VASH2$_{1-355}$_ Q122A-Fw	GAAGACCCTGGCGTACAACCACACA
VASH2$_{1-355}$_ Q122A-Rv	TGTGTGGTTGTACGCCAGGGTCTTC
VASH2$_{1-355}$_ E152A-Fw	GAGATGACAAGGGCGAGCCTGCCTATC
VASH2$_{1-355}$_ E152A-Rv	GATAGGCAGGCTCGCCCTTGTCATCTC
VASH2$_{1-355}$_ S153A-Fw	ATGACAAGGGAGGCCCTGCCTATCAAG
VASH2$_{1-355}$_ S153A-Rv	CTTGATAGGCAGGGCCTCCCTTGTCAT
VASH2$_{1-355}$_ R212A-Fw	GGCATGTCCAGGGCGGCCGAGCTGATG
VASH2$_{1-355}$_ R212A-Rv	CATCAGCTCGGCCGCCCTGGACATGCC
His-SVBP$_{1-66}$-Fw	CGCGGATCCGACCCTCCTGCCAGAAAGGAGAAGAG
His-SVBP$_{1-66}$ -Rv	CCCAAGCTTTTACTCGCCGGGGGGCTGCATCTGCTTACAAAACT
His-SVBP$_{1-66}$_K32A-Fw	CAGCAGGAGCTGGCGCAGAGACAGAGG
His-SVBP$_{1-66}$_ K32A-Rv	CCTCTGTCTCTGCGCCAGCTCCTGCTG
His-SVBP$_{1-66}$_ R34A-Fw	GAGCTGAAGCAGGCACAGAGGGCCGAG
His-SVBP$_{1-66}$_ R34A-Rv	CTCGGCCCTCTGTGCCTGCTTCAGCTC
His-SVBP$_{1-66}$_ Q35A-Fw	CTGAAGCAGAGAGCGAGGGCCGAGATC
His-SVBP$_{1-66}$_ Q35A-Rv	GATCTCGGCCCTCGCTCTCTGCTTCAG
His-SVBP$_{1-66}$_ R36A-Fw	AAGCAGAGACAGGCGGCCGAGATCTAC
His-SVBP$_{1-66}$_ R36A-Rv	GTAGATCTCGGCCGCCTGTCTCTGCTT
His-SVBP$_{1-66}$_ Y40A-Fw	AGGGCCGAGATCGCCGCCCTGAACAGA
His-SVBP$_{1-66}$_ Y40A-Rv	TCTGTTCAGGGCGGCGATCTCGGCCCT
His-SVBP$_{1-66}$_ N43A-Fw	ATCTACGCCCTGGCCAGAGTGATGAC
His-SVBP$_{1-66}$_ N43A-Rv	GGTCATCACTCTGGCCAGGGCGTAGAT
His-SVBP$_{1-66}$_ E50A-Fw	ATGACCGAGCTGGCGCAGCAGCAGTTC
His-SVBP$_{1-66}$_ E50A-Rv	GAACTGCTGCTGCGCCAGCTCGGTCAT
His-SVBP$_{1-66}$_ R34A/R36A-Fw	CTGAAGCAGGAACAGGAGGCCGAGATC
His-SVBP$_{1-66}$_ R34A/R36A-Rv	GATCTCGGCCTCCTGTTCCTGCTTCAG
His-SVBP$_{1-66}$_Y40A/N43A-Fw	GCCGAGATCGCCGCCCTGGCCAGAGTGATG
His-SVBP$_{1-66}$_Y40A/N43A-Rv	CATCACTCTGGCCAGGGCGGCGATCTCGGC

Fw and Rv represent a forward primer and a reverse primer, respectively.
